# Novel systems for the application of isolated tensile, compressive, and shearing stimulation of distraction callus tissue

**DOI:** 10.1371/journal.pone.0189432

**Published:** 2017-12-11

**Authors:** Nicholaus Meyers, Julian Schülke, Anita Ignatius, Lutz Claes

**Affiliations:** Institute of Orthopedic Research and Biomechanics, Center of Musculoskeletal Research Ulm, University Hospital Ulm, Ulm, Baden-Württemberg, Germany; University of Zaragoza, SPAIN

## Abstract

**Background:**

Distraction osteogenesis is a procedure widely used for the correction of large bone defects. However, a high complication rate persists, likely due to insufficient stability during maturation. Numerical fracture healing models predict bone regeneration under different mechanical conditions allowing fixation stiffness optimization. However, most models apply a linear elastic material law inappropriate for the transient stresses/strains present during limb lengthening or segment transport. They are also often validated using *in vivo* osteotomy models lacking precise mechanical regulation due to the unavoidable stimulation of secondary interfragmentary motion during ambulation under finitely stiff fixation. Therefore, in order to create a robust numerical model of distraction osteogenesis, it is necessary to both characterize the new tissue’s viscoelasticity during distraction and determine the influence of strictly isolated stimulation in each loading mode (tension, compression, and shear) to account for potential differences in mechanical and histological response.

**Aim:**

Two electromechanical fixators with integrated load cells were designed to precisely perform and monitor *in vivo* lateral distraction and isolated stimulation in sheep tibiae using a mobile, hydroxyapatite-coated titanium plate. The novel surgical procedure circumvents osteotomy, eliminating the undesirable and unquantifiable mechanical stimulation during ambulation.

**Methods:**

After a 10-day post-surgery latency period, two 0.275 mm distraction steps were performed daily for 10 days. The load cell collected data before, during, and after each distraction step and was terminated after no less than one minute from the time of distraction. A 7-day consolidation period separated the distraction phase and 18-day stimulation phase. Stimulation was carried out in isolated tension, compression, or shear while recording force/time data. Each stimulation session consisted of 120 cycles with a magnitude of either 0.1 mm or 0.6 mm in the tension and compression groups and 1.0 mm in the shear group. The animals were euthanized after a 3-day holding period following stimulation.

**Results:**

Our initial results show that the tissue progressively stiffens and maintains an increasingly large residual traction. The force curves during compressive stimulation show a progressive drift from compression toward tension. We hypothesize that this behavior may be due to the preferential flow of fluid outward from the tissue and a greater resistance to reabsorption during the plate’s return to the starting position.

## Introduction

Distraction osteogenesis (DO) is a surgical procedure widely used for the treatment of pathological conditions requiring the formation of a large volume of new bone. DO procedures are frequently used to correct congenital and post-traumatic limb length discrepancy and deformity, regenerate surgically excised diseased tissue, and to correct maxillofacial deficiencies. This technique is typically performed through the creation and gradual separation of a transverse osteotomy or corticotomy. The tensile stimulus of distraction initiates the differentiation of mesenchymal tissue within the distraction gap [1, 2]. Upon reaching the intended distraction length, the callus is still soft and must be held stable with appropriate fixation stiffness to allow maturation and consolidation, a period generally requiring twice as much time as distraction. Therefore, DO procedures typically require many months.

Despite technical improvement, a complication rate of approximately 10–33% persists [3, 4]. Due to the demonstrably strong osteogenic potential of the tissue tension produced during distraction[1, 2, 5, 6], it is assumed to be the interfragmentary motion during the lengthy post-distraction consolidation period which critically governs the maturation of the newly formed tissue into a stable hard callus, delayed union, or even hypertrophic nonunion. In order to improve understanding and ultimately the clinical outcome of distraction osteogenesis, it is then vital to associate post-distraction interfragmentary motion with histological development in the healing region. Furthermore, it has been shown that axial interfragmentary motion during fracture healing results in a different tissue response than a shearing motion of equal magnitude in fracture healing studies [7–11]. Consequently, it is also of great importance to characterize the tissue response to cyclic motions of different modes (tension, compression, and shear). In order to improve post-operative and specifically post-distraction patient care, understanding the influence of fixation stiffness on the consolidation of distraction callus is critical.

Through *in silico* experimentation, researchers may calculate these effects under various clinical conditions. This makes numerical models powerful tools, facilitating progress towards optimization of distraction protocols and fixation technology. Although several models of fracture healing have been developed [11–20], fewer models have sought to encompass the more complicated DO process [18, 21–25], and in particular, do not investigate the influence of mechanical stability during the consolidation phase.

Currently, the simulation of DO is greatly limited by *a priori* tissue material property assumptions and simplifications. In addition, the mechanobiological tissue differentiation rules responsible for translating the mathematical results of finite element analysis into tissue type and distribution predictions are based on *in vivo* experiments that have not precisely enough controlled the mechanical conditions within the healing region [26]. Establishing a mathematical description that approximates the in vivo, time-dependent stress-strain profile to a step-wise lengthening is foundational to computationally replicating the distraction process. This is a necessary step for the apposite implementation of the mechanobiological tissue differentiation hypothesis underlying the numerical model. Precise measurement of the viscoelastic behavior and temporal evolution of the forming callus tissue is necessary and can be accomplished with a suitable *in vivo* method to apply a pure distraction process and simultaneously record the tissue response throughout the procedure.

Two electromechanical fixators were designed to precisely perform such a controlled distraction and subsequently apply independent tensile, compressive, or shearing stimulation, simulating fixation stiffness. These fixators work in concert with a unique ovine tibia lateral callus distraction model which maintains the mechanical competence of the bone by circumventing the traditional osteotomy [27]. The novel data that these fixators generate will be useful for the calibration of currently uncertain *in silico* material parameters for an existing model of lateral callus distraction [28] as well as provide histological data in response to isolated stimulation of different magnitudes during the consolidation phase. Such histological data can provide insight into the fundamental mechanobiology of DO and further help calibrate numerical predictions.

## Materials and methods

### Experimental protocol

**Fig 1:**
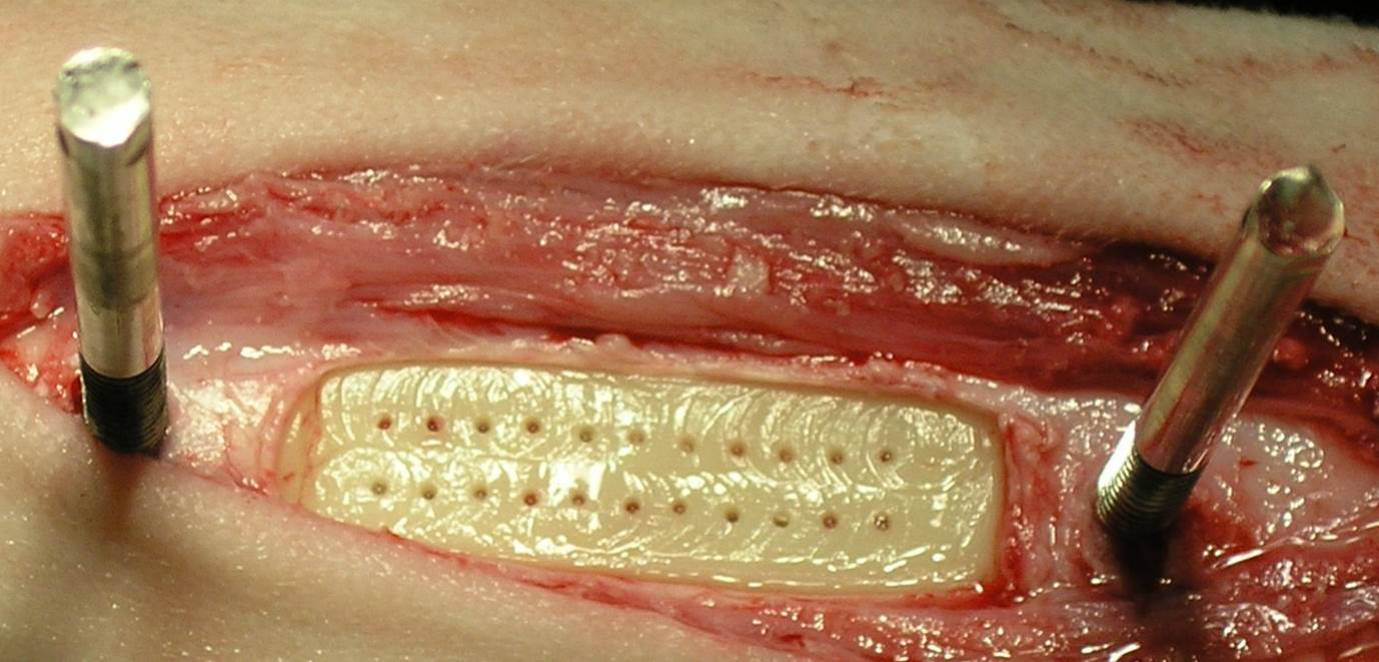
Prepared bone surface with transcortical bore holes and Schanz screws for fixator mounting.

The surgical procedure is identical to that described by Claes et al. [27]. Briefly, a 10x35 mm flat plane is milled into the anteromedial surface of the tibia and 22 Ø1.1 mm transcortical holes are drilled into the surface in two rows to allow angiogenesis to proceed from the medullary canal (Fig 1). The distraction device is attached to Schanz screws straddling the milled area and the distraction plate is set against the bone surface. All interventions on living animals were performed under isoflurane anesthesia and according to the regulations of EU Directive 2010/63/EU for animal experiments and conformed to ARRIVE guidelines (Animal Research: Reporting of in Vivo Experiments). The experiments were approved by the local ethics committee (Registration no. 1168, Regierungspräsidium Tübingen, Germany).

Following a 10-day post-surgery latency period, two 0.275 mm distraction steps are performed daily for 10 days resulting in a 5.5 mm distraction gap. For the sheep fitted with an “axial stimulation fixator,” an integrated load cell collects force data. Data collection begins prior to the distraction step to establish the steady-state tensile load remaining from the previous distraction step. Data collection continues during the distraction process and then for an additional one minute or until the decay in the force measurement is no longer readily apparent, whichever is longer. This data will be used to form a time-dependent model of the viscoelastic properties of the regenerate tissue, a critical step providing information necessary for the calibration of a predictive numerical simulation. A 7-day consolidation period follows the 10-day distraction phase during which no stimulation is applied.

Following the consolidation period, an 18-day stimulation phase begins during which the tissue regenerate is subjected to isolated cyclic compression, tension, or unidirectional shear. Two magnitudes of stimulation (0.1 mm and 0.6 mm) have been selected for the tensile and compressive stimulation groups, and one magnitude was chosen for the shear stimulation group (1.0 mm). The magnitudes were chosen based on an existing tissue differentiation hypothesis predicting intramembranous bone formation for small strain values and compressive hydrostatic pressure (ε < 5%, P < 0.15 MPa) and endochondral ossification at larger strain values and compressive hydrostatic pressure (ε > 10%, P > 0.20 MPa) [15]. Each group received 120 stimulation cycles per day with a frequency of 2 Hz (60 seconds of stimulation), 0.33 Hz (360 seconds of stimulation), or 0.2 Hz (600 seconds of stimulation) for stimulation magnitudes of 0.1 mm, 0.6 mm, and 1.0 mm respectively.

In order to properly compare tissue response during stimulation, a similar strain rate between groups is necessary. The literature indicates that the number of stimulation cycles plays an important role in bone formation [29, 30]. Furthermore, an *in vitro* study indicates that cell proliferation does *not* significantly depend on the frequency of cyclic strain when the cycle number is held constant [29] while an *in vivo* study on sheep tibiae shows that an axial stimulation controlling for magnitude and total number of cycles does *not* show any influence on the healing for frequencies of 1, 5, and 10 Hz [9]. Another study has reported that the strain rate *does* play a role in the modulation of healing within fracture models [31]. We therefore determined it would be most appropriate to control for the total number of cycles and initial strain rate by maintaining a constant plate velocity between groups of different amplitudes. The combination of frequency and time was calculated to produce the same distraction plate velocity during stimulation (0.4 mm/s), and thereby a consistent initial strain rate between stimulation groups. The selected stimulation protocol parameters should then produce the most comparable biological and mechanical results.

Following the stimulation phase, the animals were held for an additional three days before euthanasia by penetrating captive bolt and exsanguination.

### Fixator construction

One fixator was designed to collect *in vivo* tissue relaxation data during a highly reproducible, electromechanically controlled distraction procedure and subsequently apply either tensile or compressive stimulation of a selected magnitude, frequency, and time. This fixator is herein designated the “axial stimulation fixator.” A second fixator was developed to provide shearing stimulation following a manual, mechanical distraction nearly identical to the method used by Claes *et al*. [27]; this design is identified as the “shear stimulation fixator.” This piecemeal design was chosen to minimize the size, weight, and complexity of the fixators relative to an electromechanically controlled, multi-directional system so as not to interfere with the animal’s normal activity nor pose an appreciable risk of injury during ambulation.

#### Axial Stimulation Fixator

**Fig 2:**
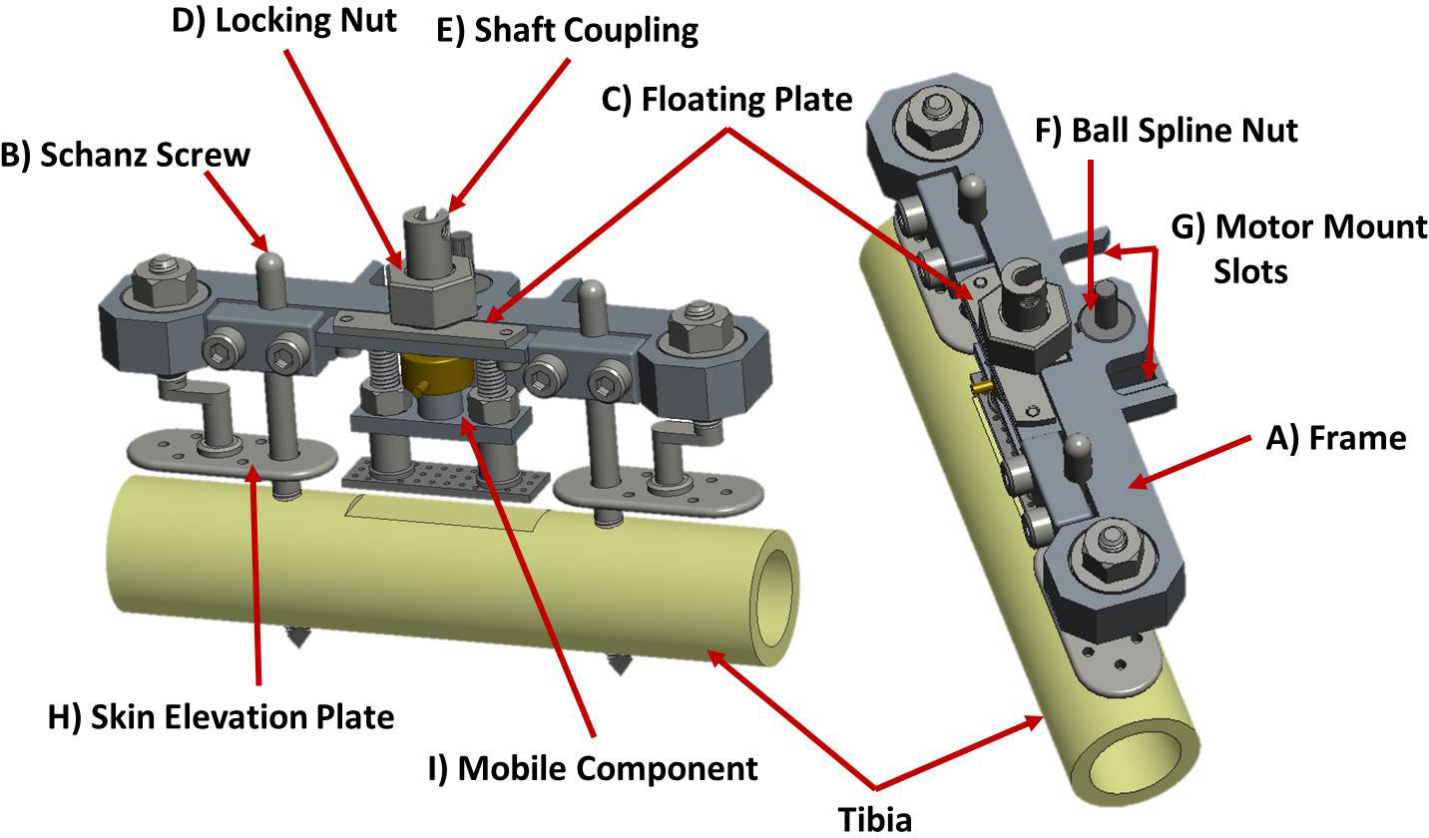
Model of mounted axial stimulation fixator.

**Fig 3:**
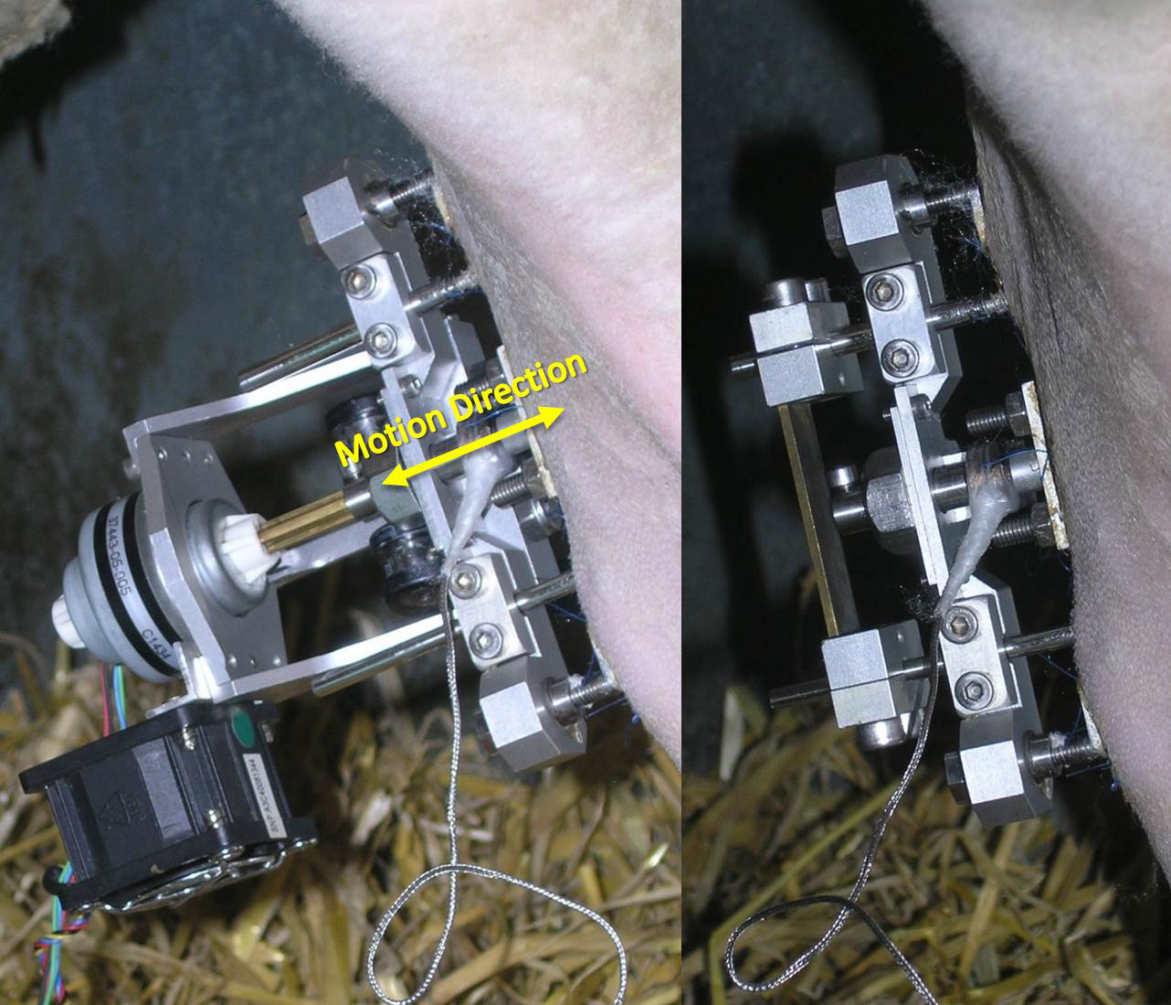
Axial stimulation fixator mounted *in vivo*. Left) Fixator mounted *in vivo* with linear actuator attached for distraction or stimulation. Right) Fixator with motor removed and protection plate mounted.

Figs 2 and 3 depict a model of the axial stimulation fixator with labeled components and the system mounted *in vivo*, respectively.

The axial stimulation fixator consists of a frame (Fig 2A), milled from a single block of aluminum, which is mounted to a stainless steel Ø5 mm proximal Schanz screw and Ø4 mm distal Schanz screw (Fig 2B) (DePuy Synthes Trauma, West Chester, PA, USA) using two small clamps each held by two standard, steel, M4 screws. Since the mechanical competence of the bone is maintained by eliminating the osteotomy, the frame need not bear load and was therefore designed to be low profile and lightweight; the full fixator assembly (without stepper motor) weighs only 210 grams. Overall fixator dimensions are provided in a supplementary figure (S1 Fig). A protection plate attaches to the Schanz screws above the shaft coupling to prevent the mobile segment from direct external contact (Fig 3).

A stainless steel component containing a concentric clamping mechanism, hereafter referred to as the floating plate (Fig 2C), is screwed to the top of the frame; the clamp is coaxial with a larger hole through the fixator when mounted. The concentric clamping mechanism is used to maintain the distraction height. When locked with a complementary custom nut (Fig 2D), the clamp applies symmetrical pressure to a shaft coupling passing through the fixator (Fig 2E) which connects the distraction plate to the linear actuator. This minimizes the forces and moments transferred to the load cell inherent during any locking procedure. The independence of the clamping component allows a close running fit between the shaft coupling and the clamp without increasing friction during motion. This is achieved by loosening the floating plate screws thereby allowing the clamp to passively shift within a Ø0.2 mm tolerance zone within the oversized hole through the fixator. This prevents binding of the shaft coupling within the small clearance of the clamp during distraction or stimulatory motion under the harsh conditions of *in vivo* experimentation. An SSPS4 stainless steel ball spline nut (Fig 2F) (Nippon Bearing Co., Ltd., Ojiya-City, Niigata, Japan) is secured into the frame parallel to the clamping mechanism along the width of the frame to receive a rail. The rear side of the frame features two slots (Fig 2G) to which a custom designed motor bracket may be attached. The motor shaft enters a slot cut into the shaft coupling and quick release lever clamps provide a simple and rapid method of securing the motor bracket. This allows the motor to be removed and reproducibly located between distraction or stimulation sessions, greatly reducing the overall size and weight of the fixator. Additionally each end of the frame holds a nut used to raise a subcutaneous, stainless steel plate (Fig 2H). These plates track over each Schanz screw and are used to elevate the skin above the distraction site so that skin tension cannot influence the measurements taken during distraction and relaxation or stimulation.

**Fig 4:**
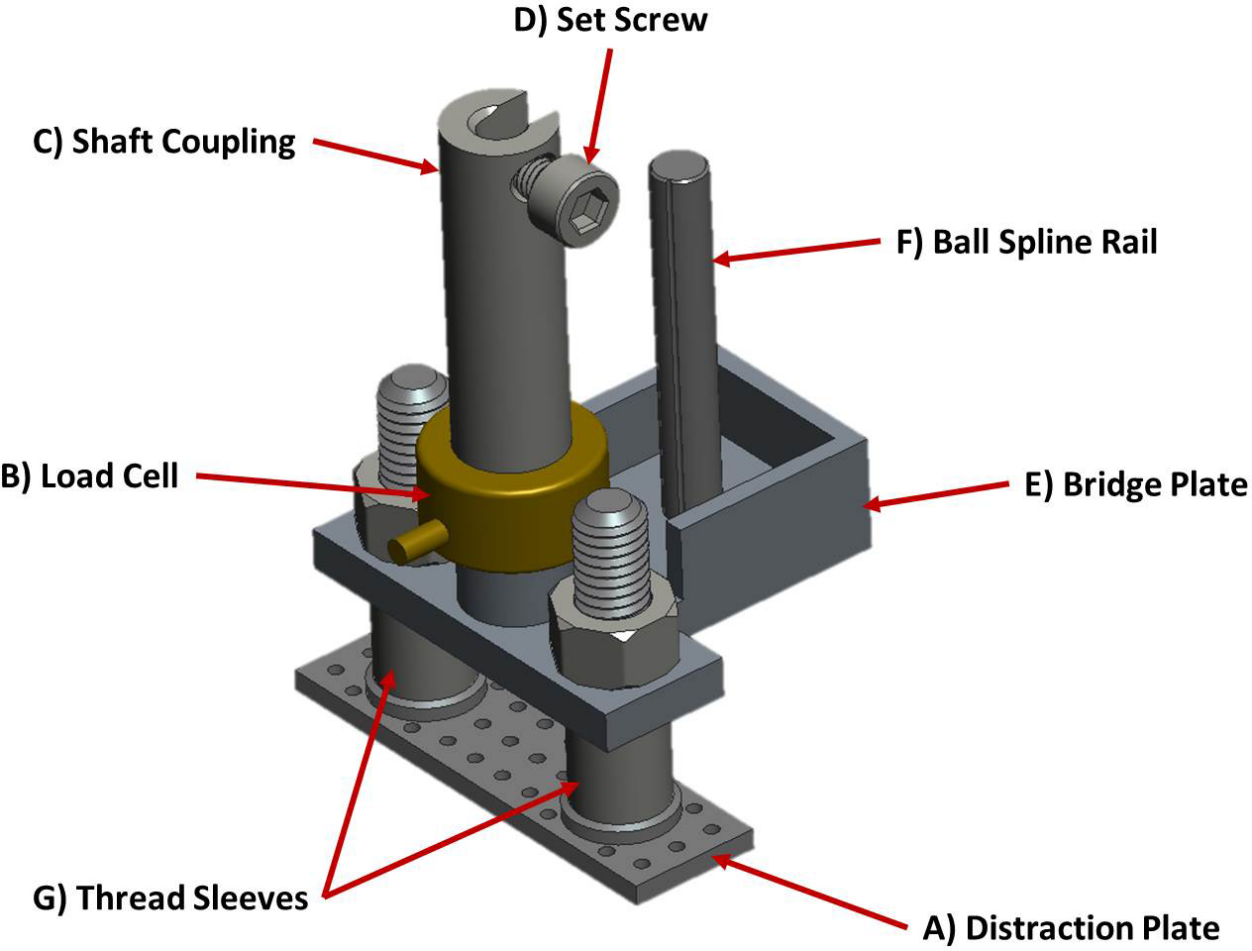
Model of axial fixator bridge assembly.

The mobile component of this fixation system (Fig 4) is designated the bridge assembly. The bridge assembly links the hydroxyapatite-coated, titanium distraction plate (Fig 4A) (CelGen AG, Zug, Switzerland), a Burster 8411–200 subminiature load cell (Fig 4B) (Burster Praezisionsmesstechnik GmbH & Co. KG, Gernsbach, Germany), and the stainless steel shaft coupling (Fig 2E, Fig 4C), containing a set screw to secure the motor shaft (Fig 4D), in series with an aluminum component referred to as the bridge plate (Fig 4E).

The titanium distraction plate has a 10 x 35 mm rectangular footprint and is perforated to allow free nutrient exchange across its surface. Two threaded Ø5 mm posts are laser welded to the top surface of the distraction plate and stand 20 mm apart. A stainless steel, 8 mm-tall, Ø7 mm sleeve is threaded over each of these posts to reduce friction at the skin as well as the likelihood of infection from debris and pathogens that would otherwise enter the threads of the distraction plate. The bridge plate sits atop these sleeves and is securely locked with standard Ø5 mm nuts. The load cell body measures only Ø12.7 mm and 7.4 mm tall (excluding the threaded connections). Therefore, using one of the M3 external threads it can be secured directly into the top of the bridge plate situated between the threaded posts of the distraction plate. The shaft coupling is threaded to the top connection of the load cell and passes through the floating plate in the middle of the fixator frame. The placement of the load cell beneath the shaft coupling eliminates any influence of friction between the shaft coupling and floating plate from the force measurements.

Parallel to these components, within the plane perpendicular to the distraction plate and press-fit into the bridge plate is the complementary ball spline rail (Fig 4F). This particular linear bearing option resists rotation up to 1.05 Nm around the guiding axis, preventing the shaft coupling and load cell from experiencing a lateral bending load under the free-hanging weight of the bridge assembly while mounted to the tibia of the sheep. Three walls of the bridge plate are elevated around the ball spline rail to help shield body fluids from entering the linear bearing.

#### Shear stimulation fixator

**Fig 5:**
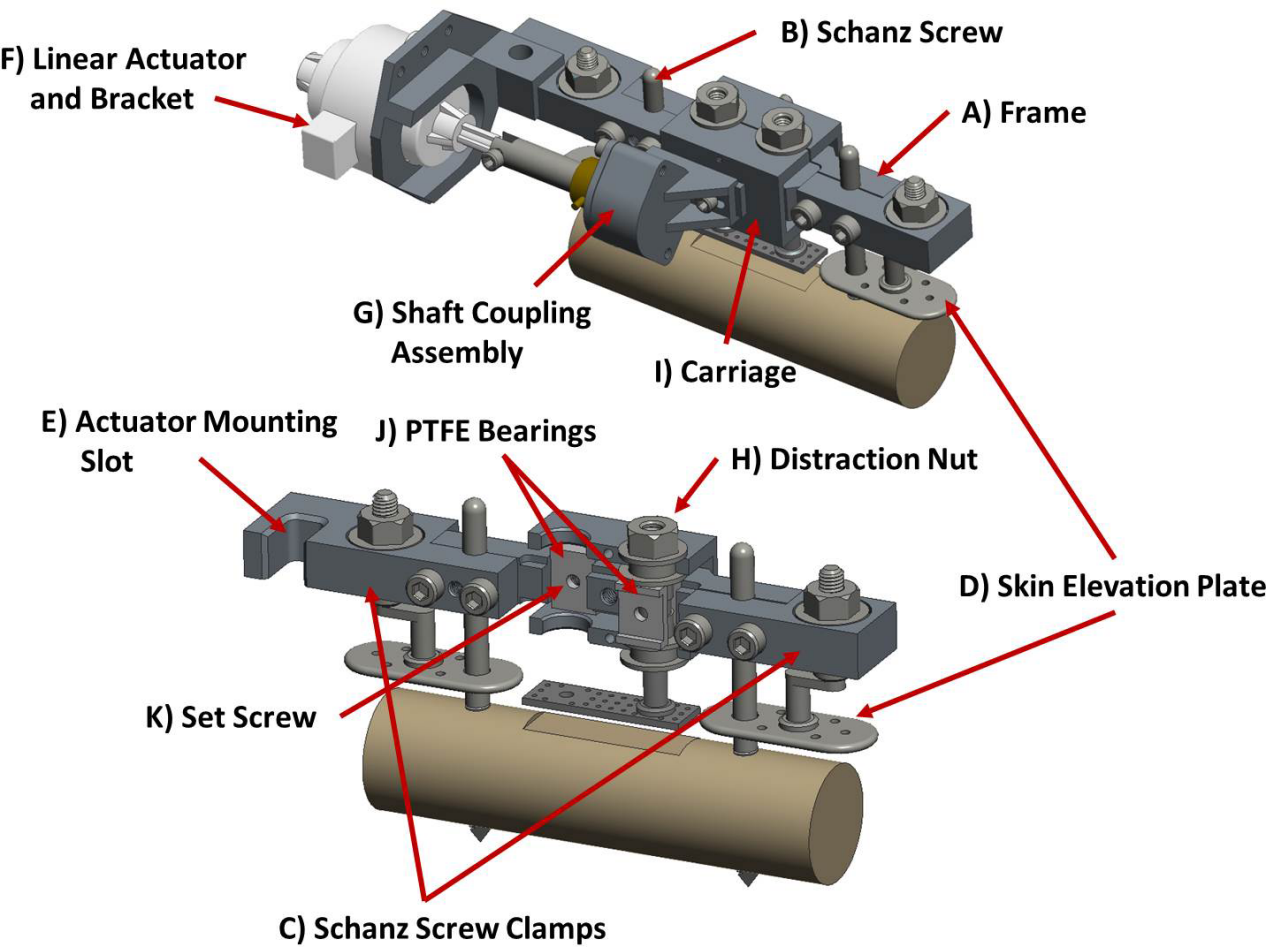
Model of mounted shear stimulation fixator with components removed to demonstrate internal features. Top) Complete shear stimulation fixator with mounted linear actuator and shaft coupling assembly. Bottom) Fixator with frame components removed to demonstrate internal PTFE bearing configuration.

**Fig 6:**
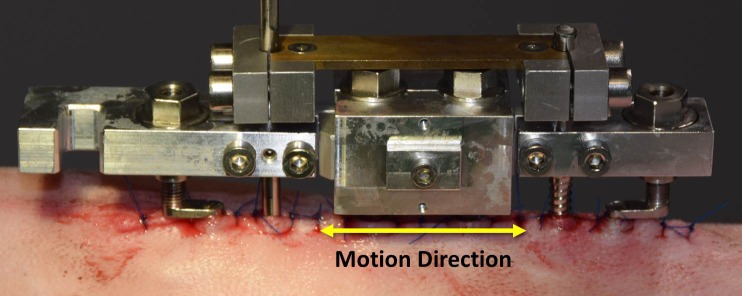
Mounted shear stimulation fixator. Fixator mounted *in vivo* with protection plate.

Figs 5 and 6 depict a model of the shear stimulation fixator with labeled components and the system mounted *in vivo*, respectively.

The frame (Fig 5A) is mounted to a stainless steel Ø5 mm proximal Schanz screw and Ø4 mm distal Schanz (Fig 5B) screw in the same manner as the axial stimulation fixator; two clamps (Fig 5C), each held by two standard, steel, M4 screws, are used to secure the frame to the Schanz screws. Unlike the axial stimulation fixator the clamps of the shear stimulation fixator contain the distraction nuts for the skin elevation plates (Fig 5D). Additionally, the distal clamp contains the mounting slot (Fig 5E) for the stepper motor/linear actuator (Fig 5F) used to apply the cyclic shearing motion. The entire fixator assembly (without linear actuator or shaft coupling assembly) weighs 225 grams. Overall fixator dimensions are provided in a supplementary figure (S2 Fig).

The load cell is incorporated into a custom-designed, removable shaft coupling assembly (Fig 5G), which allows radial positioning adjustment within a Ø2 mm zone. This coupling improves alignment with the stepper motor/linear actuator to reduce bending loads transferred to the load cell. For this fixator, distraction is achieved using custom distraction nuts (Fig 5H) which are connected by a two-component “carriage” (Fig 5I). This rigid connection maintains the parallel alignment of the threaded posts of the distraction plate thereby preventing deflection and the associated loss of stimulatory motion under the eccentric lateral load during shear stimulation. This carriage also serves as the point of connection for the shaft coupling assembly. The load cell used for the shear stimulation fixator is the same as those used for the axial stimulation fixator, but it is situated perpendicular to the direction of distraction (Fig 6) in order to measure the force-displacement relationship during stimulation. The two aluminum components forming the main body of the shear stimulation fixator frame contain rectangular slots that hold polytetrafluoroethylene (PTFE) inserts (Fig 5J). These inserts act as bearing surfaces to allow low-friction, lateral translational motion of the custom distraction nuts. In the rear of the fixator are two M3 set screws (Fig 5K). The PTFE inserts contain a central hole through which these set screws may pass and impinge upon the distraction nuts. This both prevents the accidental rotation of the nuts between distraction steps and locks the lateral translational motion between stimulation sessions.

#### Electronics and software

**Fig 7:**
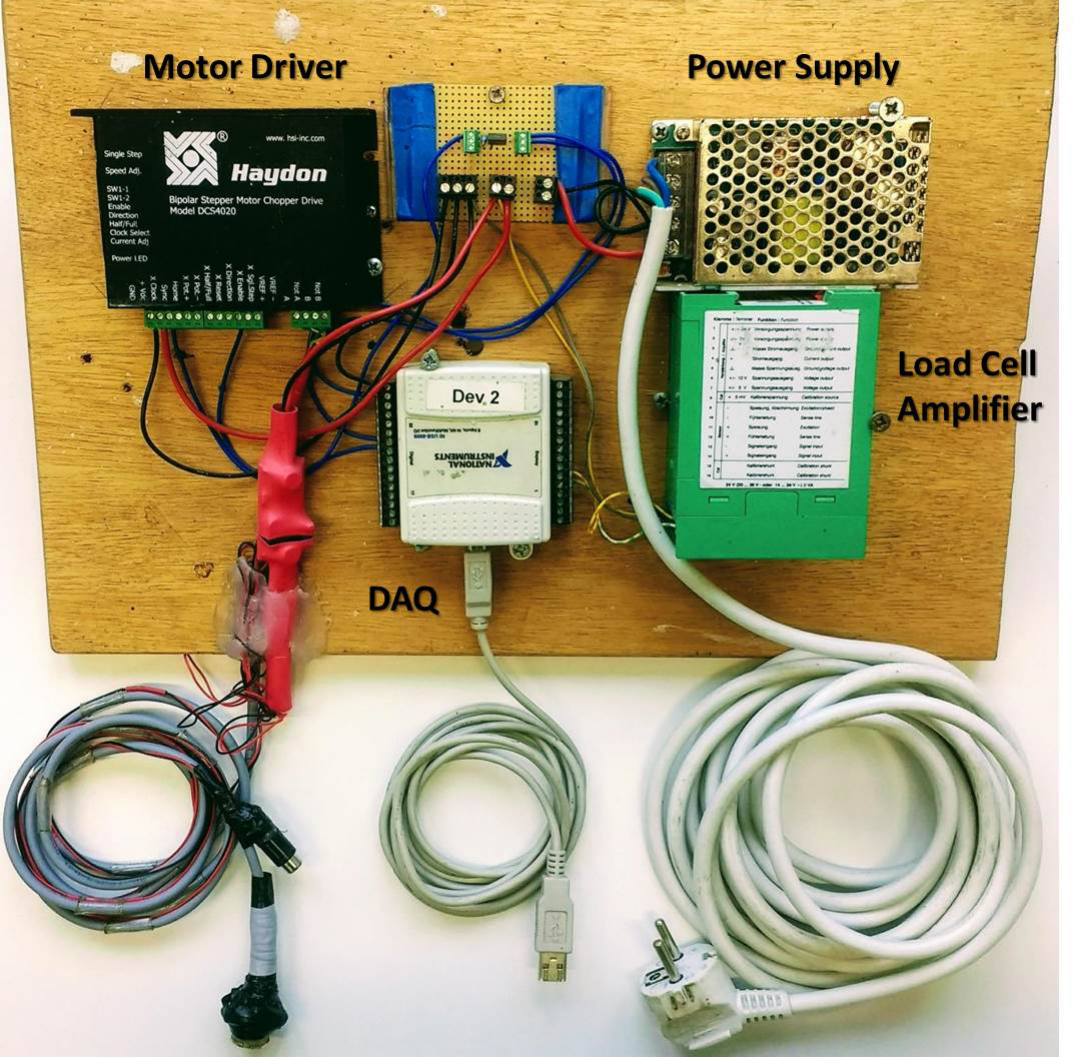
Control system hardware.

The entire fixator control system is mounted to a mobile wooden board (Fig 7). A 24 volt power supply (TRACO Electronics AG, Baar, Switzerland) powers a bipolar chopper motor driver (Haydon Kerk Motion Solutions Inc., Waterbury, CT, USA) and a load cell amplifier module (Burster Praezisionsmesstechnik GmbH & Co. KG, Gernsbach, Germany). As previously mentioned, the load cells used are subminiature Burster 8411–200 and are capable of measuring up to 200N in both tension and compression. A USB 6009 data acquisition (DAQ) device (National Instruments Corporation, Austin, TX, USA) connects to a notebook computer loaded with custom control software programmed in LabVIEW (National Instruments Corporation, Austin, Texas, United States). The DAQ is responsible for both transmitting the program commands to the chopper driver and collecting the output from the load cell amplifier for displaying and recording force data.

The chopper driver powers a custom, NEMA size 14, Haydon Kerk hybrid stepper motor/ linear actuator. The stepper motor is solid shimmed to prevent passive axial motion of the shaft under high loads and has a linear resolution of 0.0121 mm/step. The captive configuration provides protection of the internal transmission against the harsh *in vivo* operating conditions. The 12.7 mm stroke length minimizes the overall dimensions while providing sufficient range for distraction and subsequent stimulation. The linear actuator is capable of a peak output force of approximately 220 N, twice the necessary capability. Because the tissue is incapable of resisting with such a high load within the time period of distraction or stimulation, the high force output actuator helps ensure proper positioning despite the open-loop control.

The software allows manual control of the linear actuator for proper positioning of the shaft and the performance of each distraction step. Automatic control functions run the stimulation protocols. The software allows multiple control parameters to be set. During manual positioning this includes the speed of motion and the size of each motion step. Under automatic control, the total duration of stimulation, frequency, and stimulation magnitude are independently adjustable. The data is recorded with a sample frequency of 100 Hz and displayed in a force vs. time graph. The full data set is output to a text file for further analysis.

### System validation

#### Floating plate locking

Due to the open loop nature of the control system, benchtop tests were performed using a linear variable differential transformer (LVDT) with a resolution of 1 μm at the distraction plate surface to ensure both the distraction and stimulation phases would reproducibly perform as intended.

The holding strength of the concentric clamping mechanism was tested under progressively larger static loads, up to 98 N, hung directly from the titanium plate; at no point could slipping be detected for any of the floating plates. Once the holding strength had been verified, calibration weights were used to calibrate the load cells used in the experiment and separate calibration files were created for each load cell. The clamps were tested and the calibration procedure was performed for every load cell after each experimental group before re-use.

#### Distraction

The distraction step was then performed with progressively larger static loads up to 98 N to ensure that the linear actuator would reproducibly reach the prescribed distraction step magnitude under a resistive load; the target distraction step magnitude was consistently achieved. Furthermore, the accumulated distraction height after multiple successive distraction steps indicated that the performance of the linear actuator was consistent through the stroke length. This testing indicated that the linear actuator could reproducibly achieve the proper distraction height and the set screw used to connect the linear actuator to the shaft coupling provided sufficient holding strength.

A final test was performed in which we measured the position of the shaft coupling before and after each distraction step, and after locking the shaft coupling with the floating plate clamp. A small, sporadic loss of distraction height due to clamping could be noted.

#### Stimulation

The axial stimulation fixator was tested in both loaded and unloaded tension and compression. The tensile stimulation was simulated under a free hanging 98 N load and no slipping or drift in position could be detected over stimulation.

In the absence of literature data for these unique experimental conditions, we performed a pilot study to establish an expectation of resistance to 0.6 mm of compressive stimulation. We noted peak compressive forces up to approximately 80 N. In order to provide a more realistic benchtop test to validate the open loop control during compressive stimulation, the fixator was mounted to a wooden board and 0.6 mm compression was applied to a silicone rubber block with a thickness of 10.6 mm and a base area of 15x40 mm. The peak force at 0.6 mm was 70 N and the full-scale compressive displacement was achieved over each cycle of the stimulation protocol.

Likewise, shear motion was tested in loaded and unloaded configurations *in vitro* to determine the influence of backlash in the system. Elastic bands were used to apply a resistive preload to generate contact between mobile components as they would occur *in vivo* under the resistance of tissue. Although the resulting displacement was consistent across cycles for each fixator, the total achieved displacement varied between fixators from 0.9 mm to 1.0 mm. The fixators were disassembled, reassembled, and retested; consistent total displacement was achieved for each fixator. The fixators were then disassembled and the four PTFE bearings were randomly exchanged between fixators. Each fixator was then tested again and the resulting total displacement for each individual fixator changed but remained between 0.9 and 1.0 mm. It was also noted that the friction within each fixator varied after exchanging the bearings. We concluded that the reduction in displacement and the friction variation is due to lateral toggling of the distraction plate between the PTFE bearings as a result of the eccentric position of the linear actuator and the manner in which the bearings fit within each fixator, respectively. The bearings were subsequently matched to each fixator to provide the least friction and the total displacement for each fixator/bearing combination was tested and recorded.

### Technical procedure

#### Axial stimulation fixator

The distraction and stimulation handling procedures for the axial stimulation fixator are nearly identical and begin with the removal of the protection plate and manual measurement of the distraction height. At the beginning of the distraction phase, the skin plates are elevated above the distraction plate and are further elevated by an amount equal to the distraction step prior to each distraction to maintain a relative position above the distraction plate. Elevation of the skin plates ends with the final distraction step. For the tensile groups, adequate skin plate distraction is added to ensure the additional plate motion does not exceed the level of the skin plates. Adjustment of the skin plates is the only significant difference between the handling protocol of the distraction phase and stimulation phase. The load cell is then connected and the motor is attached. The motor shaft is electronically positioned using the manual control functions of the LabVIEW software and locked within the shaft coupling using a set screw. The nut of the floating plate is loosened to release the concentric clamping mechanism. Finally, the two floating plate screws are loosened to allow passive motion and prevent significant friction during the distraction step or stimulation session.

During the distraction phase, the manual step size is set to 0.275 mm and the data collection is initiated. After ensuring that the load cell is functioning properly and the initial tissue tension is recorded, the distraction step is performed with a plate velocity of 1 mm/s. Data collection continues until the decay of the force signal is no longer readily apparent but for no less than one minute. This ensures sufficient data for the analysis of the regenerate tissue relaxation behavior. Distraction is performed twice daily for a 10-day period.

During the stimulation phase, the proper stimulation parameters are set within the automatic control portion of the program. The data collection begins with the initiation of the automatic control and ends when the control program terminates according to the defined stimulation parameters. Stimulation is performed once daily for an 18 day period.

The mechanical handling procedure is then followed in reverse, tightening the floating plate screws, locking the shaft coupling with the floating plate nut, unlocking the motor shaft and removing the motor, and finally re-measuring the distraction height to ensure the proper distraction was achieved or maintained after stimulation following locking. If the measured distraction height varied from the expected cumulative distraction height by 0.05 mm or greater after relocking the procedure was repeated and a corrective distraction of appropriate magnitude was performed.

#### Shear stimulation fixator

The distraction procedure for the shear stimulation fixator is nearly identical to that from the predicate study of Claes et al. [27]. Twice daily, following the removal of the protection plate and elevation of the skin plates, two custom distraction screws are turned one third of a revolution to produce a 0.27 mm distraction step. The rigid link between the frame and the distraction plate precludes the incorporation of a load cell for data collection during the distraction phase for sheep in the shear stimulation group. Upon completion of the final distraction step, a set screw is deployed into the top of each distraction nut and fastened firmly against each threaded post of the distraction plate. This ensures that no play can occur between the threads of the distraction plate and the distraction nuts thereby preventing loss of stimulation magnitude during lateral motion.

During the stimulation phase, the protection plate remains in place. The load cell assembly is attached to the fixator body. The stepper motor is then attached to the distal Schanz screw clamp and manually positioned to be locked into the shaft coupling by means of a set screw. Once the motor shaft and load cell have been rigidly connected, the posterior set screws are loosened to allow lateral motion. As with the axial stimulation fixator, the stimulation parameters are set within the automatic control block of the LabVIEW software and initiated. The data collection begins with the program and terminates automatically once the stimulation parameters have been fulfilled.

## Preliminary results

The following preliminary results are based on the completed experiments of a subset of our planned study. The results presented herein are not intended to represent the results of our experimental work. As such, the presented measures have not yet been statistically analyzed. The values presented are intended only to demonstrate the capabilities of the experimental set-up and hint at general trends in tissue behavior.

### Peak distraction forces

**Fig 8:**
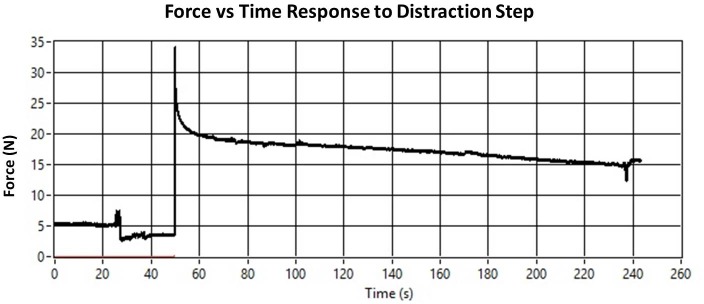
Example relaxation curve taken during distraction. Sample of data collection before, during, and after distraction on the 8^th^ day of the 10-day distraction phase. Spikes in the data are the result of sheep motion during data collection.

An average peak force of approximately 30–35 N is achieved consistently throughout the distraction process with a range between 20 and 40 N (Fig 8).

### Residual traction

The steady-state residual traction, collected before each further distraction step, increases from 0 N after the first distraction to approximately 15 N at the end of the distraction phase (Fig 8).

### Relaxation behavior

Our initial force results, capturing the relaxation process of the forming callus over the distraction period, have been fit with biphasic exponential functions for each of three preliminary sheep and for each of the 20 individual distraction steps. However, we have found that there is great variability among the fit parameters for the small sample analyzed for first results. No discernible evolutionary pattern across the distraction phase could be detected when individual distraction force relaxation curves were analyzed.

### Stimulation

During our first experiments using the shear stimulation fixator, excessive noise and artifacts resulted from the eccentric positioning of the load cell, sheep motion during data collection, and varying levels of inherent friction in each fixator. For this reason, no preliminary data has been presented for specimens of the shearing stimulation group. Once the experiments have been completed, the force data will be analyzed to determine their utility and reported in future work. Herein, we discuss only the preliminary mechanical results of the large amplitude tension and compression stimulation groups.

**Fig 9:**
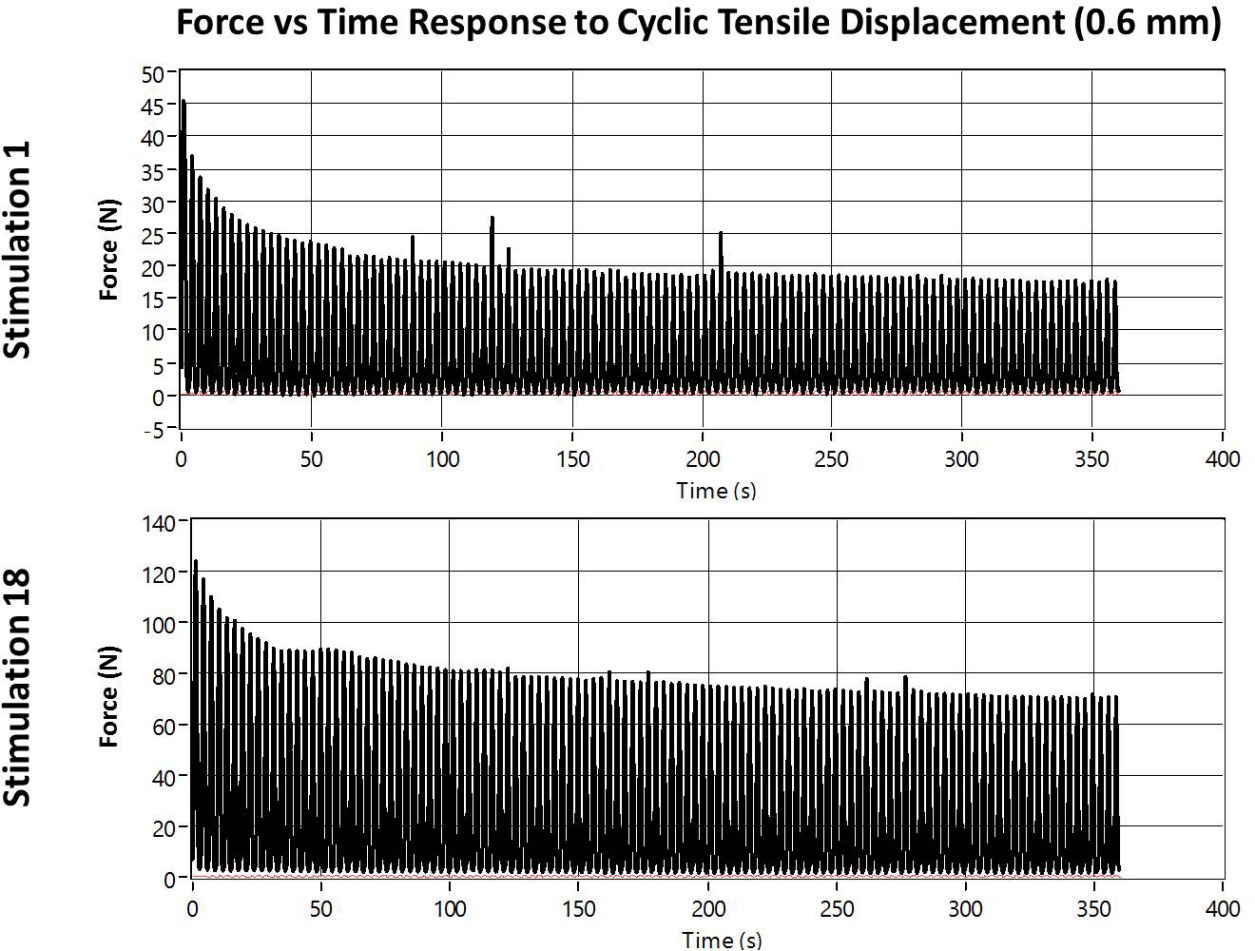
Example large amplitude tensile stimulation. Top) Stimulation session 1. Bottom) Stimulation session 18.

Our initial large amplitude tensile stimulation curves indicate an increasing tensile load as the stimulation reaches its peak tensile displacement of 0.6 mm and decreases back to 0 N as the cycle returns to the starting position. The peak forces at the beginning of stimulation reach 45 N and decay to approximately half over the remaining stimulation cycles. By the end of the stimulation phase, the peak forces have increased dramatically, up to approximately 120 N, and subsequently decay to 70 N over subsequent cycles (Fig 9).

**Fig 10:**
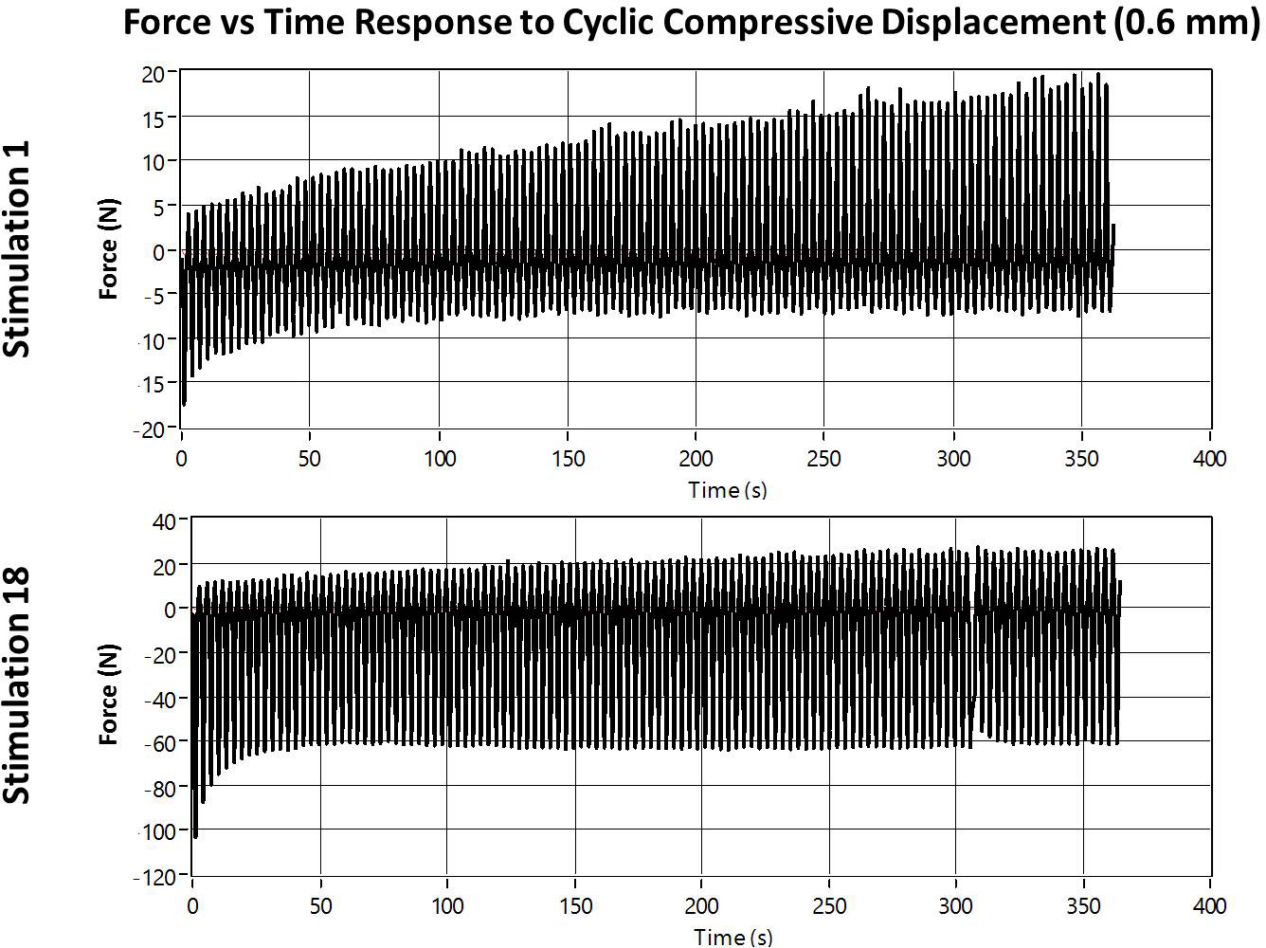
Example large amplitude compressive stimulation. Top) Stimulation session 1. Bottom) Stimulation session 18.

The initial large compressive stimulation force curves demonstrate a more complicated response to the cyclic stimulation. During the first cycle, a peak compressive force is achieved as the full 0.6 mm compressive displacement is reached. Over subsequent stimulation cycles, the peak compressive load decays while a tensile load develops as each stimulation cycle ends and the distraction plate returns to the initial position (Fig 10). The overall affect appears as a drifting of the force curve from purely compressive to each cycle split in some proportion between compressive and tensile loading.

## Discussion

To date, distraction osteogenesis research has been limited by incomplete understanding of the material properties of the newly developing gap tissue and its evolution throughout the distraction and consolidation period. This shortcoming has inhibited the development of predictive models that could be used to optimize patient care through improved technical protocols and fixation hardware. Furthermore, the inability to control precisely the mechanics at an osteotomy/corticotomy has prevented researchers from uncovering the specific influence of isolated stimulation modes and magnitudes on tissue differentiation. This work has provided an *in vivo* response to these limitations, allowing precise mechanical conditions and measurements throughout the entire procedure.

The combination of these novel fixator systems and surgical procedure allows the measurement of the viscoelastic response of the newly forming tissue at every step of the distraction phase. This data will be used to estimate the complex, time-dependent constitutive relationship to characterize the tissue’s progression and improve predictive numerical models of distraction osteogenesis. Subsequently, isolated stimulation in tension, compression, or shear can be applied while continuously recording force measurements as a function of time and displacement. The histological response to these loading modes and magnitudes will improve our understanding of the mechanobiology and provide insight into the most critical mechanical parameters necessary for reducing complication frequency and healing time for DO patients.

In addition, these devices could be used to test a wide array of experimental protocols to isolate the influence of different clinical parameters. Such experiments could help to identify generally optimal treatment limits. Improvements in outcome could be analyzed for the length of the latency phase, distraction phase variables, and 3-dimensional fixation stiffness leading to the most beneficial stimulation during consolidation.

### Peak distraction forces

At first glance, the low range in average peak forces might suggest that the fibrous soft tissue is not changing significantly or rapidly. However, it is essential to recognize that the constancy of the distraction step size leads to a continuous decrease in strain.

The strain is defined as the relative deformation and can be expressed as the ratio of the change in length (the step size) to the initial length (gap before each distraction step). The bone front does not appreciably extend during the course of the 10-day distraction phase and so the increasing gap reduces the relative percentage of deformation with respect to the height preceding each distraction step. This decay is faster during the first distraction steps. Since the distraction plate is placed in contact with the surface of the bone during surgery, the initial gap is zero and the strain of the first step is incalculable. The second distraction step yields a strain of 100% since the step size is equal to the initial gap for that step (the gap formed after the first distraction step). During the third distraction step, the strain has already been reduced to 50% and by the final distraction step, assuming no bone growth, the strain falls to just over 5%. This also occurs in the clinical scenario although the values of strain and the decay of strain magnitude are dependent on the protocol parameters.

Considering the decay in distraction strain, the consistency of the peak distraction forces indicates that the tissue is stiffening in a manner roughly proportional to the decrease in strain magnitude. Taking a peak force of 30–35 N, the stiffness can be estimated between 110–130 N/mm. In order to estimate the change in material properties specifically, the elastic modulus can be approximated. Assuming the callus area to be equal to the area of the distraction plate (350 mm^2^), a peak elastic modulus of 86–100 kPa at the start of distraction increased to 1.6–1.9 MPa by the end of the distraction phase. These estimations are in rough agreement with other studies which have attempted to estimate fracture and distraction callus stiffness or material properties *in vivo*. Leong and Morgan measured the stiffness of various tissues in a fracture callus by nanoindentation. They found a stiffness of 0.61–1.27 MPa for granular tissue [32]. Mora-Macías et al. have reported an instantaneous modulus between 0–15 MPa in a bone segment transport model in the ovine metatarsus [33].

When comparing these data, the tremendous difference in experimental set-ups should be noted. Neither the work of Leong and Morgan nor Mora-Macías et al. could prevent the secondary motion associated with osteotomy models under finitely stiff fixation. The resulting uncontrolled and unquantifiable interfragmentary motion may lead to a different pattern of tissue stiffening and differentiation patterns than those of the present work in which the secondary motion was eliminated by circumventing osteotomy.

Additionally, the work of Mora-Macías et al. relied on a manual distraction process, applied a much larger individual distraction step (1.0 mm vs 0.275 mm), and distracted with a different frequency, all of which would likely alter the rate and patterns of tissue evolution and the measurements taken. The slower speed of manual distraction compared to our rapid electromechanically controlled distraction makes it difficult to compare force response since viscoelastic response is dependent on strain rate. Furthermore, the manual distraction procedure introduces greater measurement error due to variability in the speed of individual distraction steps; this issue is avoided with the consistency of our actuator-controlled distraction rate.

### Residual traction

The increasing residual traction is indicative of the progressive consolidation of the callus tissue and may be a critical stimulus for further tissue growth in accordance with Ilizarov’s Tension-Stress effect [1]. The emerging ability of the tissue to maintain tensile load indicates that the viscous characteristics of the gap tissue diminish with respect to its elastic characteristics. The sustained load may indicate a reduction in viscoelastic matrix flow and therefore a prolonged deformation of the mechano-sensory cell structures. This may initiate further cellular proliferation and tissue expansion to return to a stable cellular configuration within the adapted mechanostat window proposed by Frost [34]. Characterizing the progression of the tissue’s ability to maintain a tensile load after an incremental distraction is therefore vital to numerically modeling the mechanics of DO.

In comparison to the work of Mora-Macías et al. [33] who reported that the residual force consistently reached 25% or less of the peak force measurement, our preliminary results indicate that up to 50% of the traction is maintained toward the end of the distraction phase. Although, the full statistical analysis of our completed experiments may reduce the difference in experimental observations, the differences in the experimental models already mentioned may also contribute to the observed discrepancy.

### Relaxation behavior

Our subsequent, in-depth analysis of the mechanical data collected with these systems will focus on the stress-strain relationship following each distraction step. The relaxation data of each specimen will be compiled for each distraction step and used to estimate the complex, time-dependent, stress-strain constitutive relationship. The fit parameters of each distraction step will then be expressed as a function of distraction step to form a functional capable of characterizing the full evolution of the mechanical behavior throughout the distraction process. This is a necessary intermediate advancement for understanding the specific complexities of DO not found in traditional fracture healing and critical for reliably translating the mechanical situation into a biological tissue response.

### Stimulation

In the early stages of the stimulation phase, the initial compressive load and the tensile load that develops over subsequent cycles are approximately equal. However, the decay of the compressive load corresponds with an increase in tensile load up to 20–25 N. Therefore, the magnitude of tensile load exceeds the compressive load for much of the stimulation session during the early stimulation phase. As the stimulation phase progresses, larger compressive loads are registered during each compressive stroke of the linear actuator, but the tensile load continues to peak at approximately 25 N (Fig 10). This indicates that, although the tensile load always develops, the compressive load begins dominating the stimulation phase after a few days.

We hypothesize that this unusual behavior is due to the preferential flow of interstitial fluid out of the tissue during compression and a restricted ability of the fluid to re-enter as the tissue is brought back to its starting position during the second half of the stimulation cycle. As far as the authors know, this is the first time this behavior has ever been observed in an *in vivo* model of bone healing and may provide new insights into the mechanotransduction mechanisms of bone formation.

### Limitations

A primary limitation of this work is that the initial magnitudes of stimulation chosen to mimic the interfragmentary motion of ambulation under a finitely stiff fixation are based on the uncalibrated distraction osteogenesis model this very work aims to improve. This means that the model of material behavior currently implemented has not been validated. Furthermore, the model implements the current tissue differentiation hypothesis of Claes and Heigele developed for fracture healing models. This hypothesis may not remain valid; the mechanical stimulus of the distraction process is known to be a potent osteogenic stimulus and furthermore, has demonstrated a “memory effect” under which tissue development continue for at least 50 days beyond the cessation of distraction [27]. Regardless, these specifics have only been used as a starting-point and the outcome of these experiments will still provide the data necessary to characterize the viscoelastic behavior of the regenerate tissue and demonstrate the mode-dependent histological reactions that will greatly increase our understanding of the mechanobiology of mesenchymal tissue differentiation.

Although, we have seen that the amount of calcified tissue at the beginning of stimulation is negligible, the potential for interindividual differences in the rate of bone formation could lead to divergence of strain rate during the later stages of stimulation. This may influence the further growth of bone.

With respect to the shearing stimulation fixator, we recognize the following limitations: 1) varying levels of friction in each fixator due to variation in part manufacturing and surgical mounting 2) sensitivity of load cell data to the eccentric location of the load cell and actuator line of thrust relative to the line of travel of the distraction plate. These limitations lead to high variability in the recorded force data and a particular sensitivity to sheep motion during data collection. It is yet to be determined whether the force data collected during shearing stimulation will serve a useful purpose. Regardless, the proper stimulation magnitude is achieved and therefore the shear stimulation fixator fulfills its primary objective. The histological response to the shearing stimulation will provide us with the highest priority data.

## Supporting information

S1 Fig Front and side view of axial stimulation fixator (with actuator and protection plate removed) demonstrating overall dimensions. (TIF)

S2 Fig Front and side view of shear stimulation fixator (with actuator and protection plate removed) demonstrating overall dimensions. (TIF)
