# Supplementary figures and images for: Novel systems for the application of isolated tensile, compressive, and shearing stimulation of distraction callus tissue
Source: PLoS One. 2017 Dec 11;12(12):e0189432. doi: 10.1371/journal.pone.0189432 (PMC5724890; doi:10.1371/journal.pone.0189432)

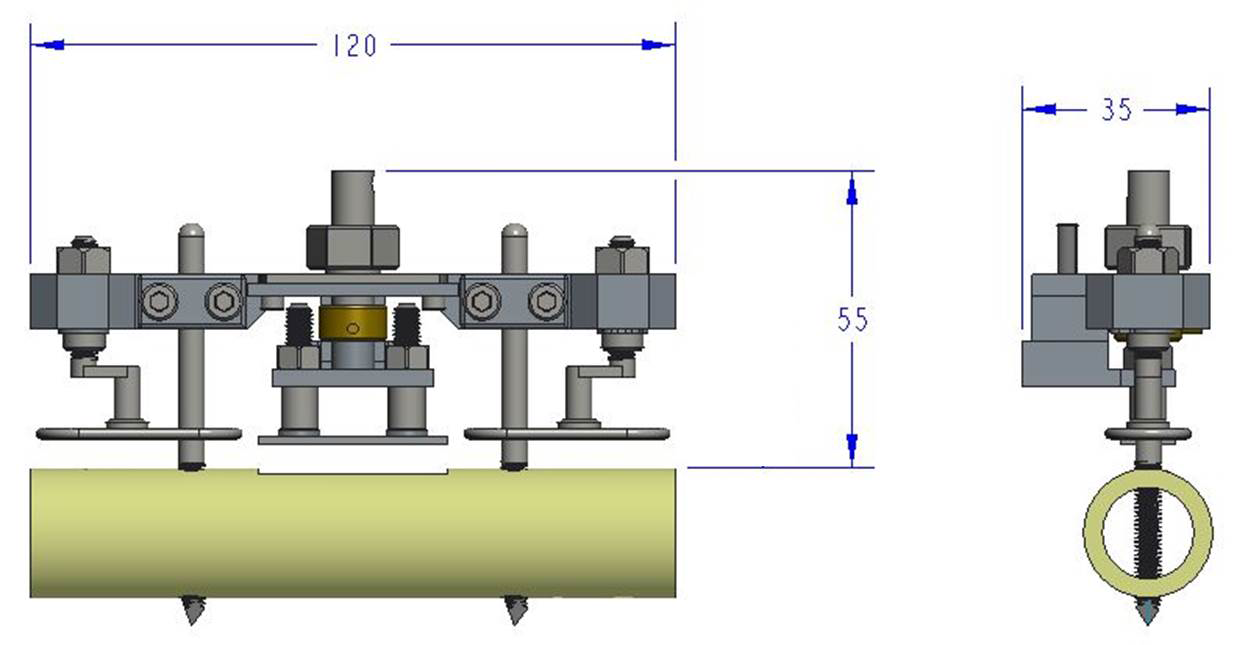

Supplement: S1 Fig — (TIF) [file pone.0189432.s001.tif]

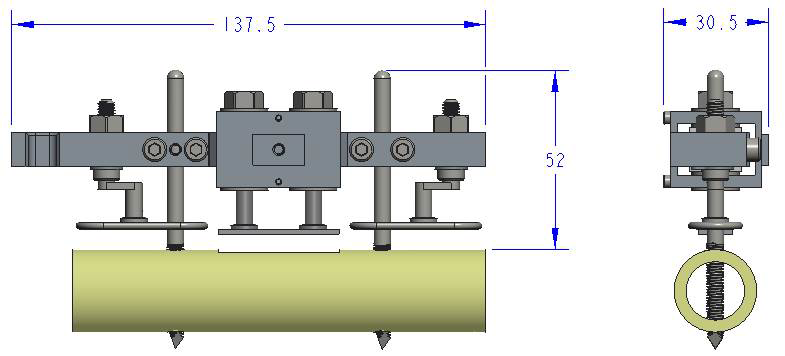

Supplement: S2 Fig — (TIF) [file pone.0189432.s002.tif]
